# Comparison of the forward and sideways locomotor patterns in children with Cerebral Palsy

**DOI:** 10.1038/s41598-023-34369-4

**Published:** 2023-05-04

**Authors:** Germana Cappellini, Francesca Sylos-Labini, Priscilla Avaltroni, Arthur H. Dewolf, Carla Assenza, Daniela Morelli, Francesco Lacquaniti, Yury Ivanenko

**Affiliations:** 1 Laboratory of Neuromotor Physiology, Istituto di Ricovero e Cura a Carattere Scientifico Fondazione Santa Lucia, 306 Via Ardeatina, 00179 Rome, Italy; 2 Department of Systems Medicine and Center of Space Biomedicine, University of Rome Tor Vergata, 00133 Rome, Italy; 3 Department of Pediatric Neurorehabilitation, Istituto di Ricovero e Cura a Carattere Scientifico Fondazione Santa Lucia, 00179 Rome, Italy

**Keywords:** Central pattern generators, Motor control, Diseases of the nervous system, Developmental disorders, Brain injuries, Neonatal brain damage, Paediatric neurological disorders, Neonatal brain damage, Movement disorders, Neurological disorders, Neurodevelopmental disorders, Neuroscience, Diseases, Brain injuries, Neonatal brain damage, Movement disorders, Neurodevelopmental disorders, Neurological disorders, Paediatric neurological disorders, Neonatal brain damage

## Abstract

Switching locomotion direction is a common task in daily life, and it has been studied extensively in healthy people. Little is known, however, about the locomotor adjustments involved in changing locomotion direction from forward (FW) to sideways (SW) in children with cerebral palsy (CP). The importance of testing the ability of children with CP in this task lies in the assessment of flexible, adaptable adjustments of locomotion as a function of the environmental context. On the one hand, the ability of a child to cope with novel task requirements may provide prognostic cues as to the chances of modifying the gait adaptively. On the other hand, challenging the child with the novel task may represent a useful rehabilitation tool to improve the locomotor performance. SW is an asymmetrical locomotor task and requires a differential control of right and left limb muscles. Here, we report the results of a cross-sectional study comparing FW and SW in 27 children with CP (17 diplegic, 10 hemiplegic, 2–10 years) and 18 age-matched typically developing (TD) children. We analyzed gait kinematics, joint moments, EMG activity of 12 pairs of bilateral muscles, and muscle modules evaluated by factorization of EMG signals. Task performance in several children with CP differed drastically from that of TD children. Only 2/3 of children with CP met the primary outcome, i.e. they succeeded to step sideways, and they often demonstrated attempts to step forward. They tended to rotate their trunk FW, cross one leg over the other, flex the knee and hip. Moreover, in contrast to TD children, children with CP often exhibited similar motor modules for FW and SW. Overall, the results reflect developmental deficits in the control of gait, bilateral coordination and adjustment of basic motor modules in children with CP. We suggest that the sideways (along with the backward) style of locomotion represents a novel rehabilitation protocol that challenges the child to cope with novel contextual requirements.

## Introduction

Although one may think about walking as a simple, straight, forward progression toward a goal, daily locomotion is not at all unidirectional. It involves frequent turns, sideways and backward steps to adjust to current environmental and social needs. In particular, children step sideways and backwards almost as frequently as they step forward^1^. They appear to choose the direction of stepping independently of immediate goals, and this has been interpreted as a key developmental strategy to explore the environment and augment the flexibility of locomotion through highly intensive practice^1^.

Exploration and flexibility of behaviour may be compromised by developmental motor disorders, resulting in more or less severe limitations of performance^2^. Therefore, it is critical to gain insights about the specific kinds of locomotor impairment associated with child pathology. There is a growing interest but limited investigations on the neural mechanisms of locomotor flexibility in motor developmental disorders. Flexibility and plasticity of neural circuits are especially important during the critical windows of development of spinal and supraspinal sensorimotor networks^3–8^. Locomotor adjustments to different task constraints typically involve multiple spinal and supraspinal pathways, as well as interactions among many subsystems. In this respect, cerebral dysfunction due to early injuries to the developing brain (such as cerebral palsy, CP) may significantly interfere with the normal engagement of supraspinal structures^9–12^. Studies of the mechanisms underlying adaptive control may also expand our knowledge about early rehabilitation strategies aimed at restoration of locomotion in young children^3,13–17^.

Neural circuits for controlling locomotion are already established in early infancy^18,19^ and their functioning is also manifested in a number of locomotor precursors^20–22^. A developmental continuity of locomotor-related movements^23^ is central for establishing independent mobility and the emergence of different gait modes^24^. In particular, changes of walking direction are common in everyday life, and can have a significant effect on children’s ability to manage the daily life. In particular, cruising (moving sideways holding onto furniture for support) represents an important developmental activity during learning to walk that infants start before the onset of independent walking^23^. Walking sideways (SW) necessitates a reorganization of muscle activity patterns^25^ and may reinforce muscles involved in the frontal plane control thus also improving balance, flexibility, and spatial awareness. Adjustments to changes in locomotor direction may require more attention and cortical resources to compensate for the impaired gait control in children with CP. In addition, muscle control abnormalities, femoral deformities, or pelvis instability in children with CP may contribute disrupting gait control when this requires a reorganization of muscle activity and specific sensorimotor adjustments following a change of direction of progression. Detailed descriptions of forward gait abnormalities in CP have been reported in numerous studies^26^. However, there is limited evidence on the mechanisms of this locomotor behavior in children with CP.

In adults, neural circuits for controlling the main form of locomotion, forward walking (FW), are relatively widely studied. Although sideways locomotion has been the subject of a smaller number of investigations, the previous studies suggest the distinct roles of the leading and trailing limb in SW and corresponding asymmetrical contribution of the musculature of both limbs and distal vs. proximal joints to the vertical force production and shock absorption^25,27^. Indeed, the specificity of SW, as opposed to other changes in the direction of progression (such as backward walking, or walking uphill and downhill), is that it is an asymmetrical task and requires a differential control and interaction between left and right limb flexor and extensor burst generators. In typically developing (TD) infants, there seem to be autonomous pattern generators for the left and right leg that interact with each other depending on the stepping conditions^28,29^. Investigating SW in individuals with CP may therefore be thought of and used as an important tool to look into impaired coordinative control of the left and right lower limbs. While difficulties in performing complex locomotor movements (walking on inclines, uneven terrain, in crowded area, climbing stairs) are included in the GMFM (Gross Motor Function Measure) assessment in persons with CP, however, directional movements, such as SW, can provide additional information about the mechanisms of impaired adaptive locomotion and possibly more comprehensive diagnosis of CP.

Here, we investigated how early injuries to the developing brain in children with CP affect locomotion when moving sideways. We hypothesized that the difficulty for children with CP to navigate sideways might be related to the lack of adjustment of the locomotor output due to the impaired cortical control and, specifically, due to the lack of flexibility in the left–right coordination of basic activation patterns of lower limbs. To this end, we studied the characteristics of the interlimb coordination during SW and FW in children with diplegia (DI) and hemiplegia (HE) from CP and typically developing children, by examining the kinematic patterns, joint moments, and spinal locomotor output associated with muscle activity of individual legs.

## Methods

### Design

This is a cross-sectional study comparing walking in two different directions, forward and sideways, between two populations of participants, children with CP and typically developing children.

Experiments were performed in the Laboratory of Neuromotor Physiology, IRCCS Santa Lucia Foundation by the gait analysis experts, in the presence of a neuro-paediatrician, physiotherapist, and one or both parents of the child. The procedure was the following. Children were asked to walk barefoot for ~ 8 m in a straight path at a self-selected speed in a large (11 m × 14 m) laboratory room. Participants were asked to walk in the following directions: forward walking (FW) and sideways walking (SW) to the left and to the right. We recorded 4–5 trials (~ 30 s each trial) in each participant for the two walking conditions (FW and SW to the left and to the right). Children did not practice SW prior to the experiment. First, we recorded forward walking trials, and then sideways walking trials. Sideways walking is defined as steps in which the leading leg is abducting and the trailing leg adducting past neutral. For SW trials, the experimenter stood in front of a child encouraging to walk and demonstrating herself/himself a few steps sideways. The experimenter initially held the child by hand, started to move sideways and asked him/her to imitate the task leaving progressively the child’s hand. The duration of the whole experiment was ~ 1 h (including placement of EMG electrodes and infrared reflective markers).

Bilateral full-body kinematics was recorded at 100 Hz by means of Vicon-Nexus system (Oxford, UK) with 10 cameras placed around the walking path. Infrared reflective markers were attached on each side of the child to the skin overlying the following landmarks: gleno-humeral joint (GH), lateral epicondyle of the elbow (Elb), ulnar process of the wrist (Wri), greater trochanter (GT), lateral femur epicondyle (LE), lateral malleolus (LM) and fifth metatarso-phalangeal joint (5MT).

Electromyographic (EMG) activity was recorded by means of surface electrodes from 24 muscles simultaneously. The following 12 muscles were recorded from each body side: gluteus maximus (GM), tensor fascia latae (TFL), adductor (ADD), rectus femoris (RF), vastus lateralis (VL), vastus medialis (VM), biceps femoris (long head) (BF), semitendinosus (ST), gastrocnemius medialis (MG), gastrocnemius lateralis (LG), soleus (SOL), and tibialis anterior (TA). All EMGs were recorded at 2000 Hz using the wireless Trigno EMG system (Delsys Inc., Boston, MA), bandwidth of 20–450 Hz, overall gain of 1000. Sampling of kinematic and EMG data were synchronized.

### Participants

This study included a sample of children diagnosed with CP and recruited from the Department of Paediatric Neurorehabilitation of Santa Lucia Foundation, aged between 1.8 and 9.9 years (Tables 1, 2). A group of TD children were also recruited, similar in age and sex (Table 3). Clinical diagnosis was based on the predominant type of motor impairment and classified according to the criteria proposed by Himmelmann et al.^30^: diplegic and hemiplegic. CP diagnosis was confirmed according to medical history, brain magnetic resonance results, and clinical examination. The Ethics Committee of Santa Lucia Foundation approved the study procedures (protocol CE/AG4/PROG.341 and CE/PROG.875) that adhered to the Declaration of Helsinki for medical research involving human participants. Informed written consent was obtained from the parents of all children (TD and CP).

**Table 1:** General characteristics of children with cerebral palsy (CP).

Characteristic of CP participants (n = 27)	
Age (year), mean ± SD	4.4 ± 2.1
Gender, n males (%)	16 (60%)
Type of cerebral palsy	
Hemiplegia, n (%)	33.3%
Quadriplegia, n (%)	0%
Diplegia, n (%)	66.7%
GMFC-s, n (%)	
Level I	66.7%
Level II	33.3%
Level III	0%

*GMFC-s* Gross Motor Function Classification System.

**Table 2:** Individual characteristics of children with cerebral palsy (CP) and performed trials.

Subjects	TCP	Side	Gender	Age, years	Lesion characteristics						GA, week	BW, g	WO, month	GMFC-s	GMFM	MAS (L/R)	SW		FW	
																	Strides, n	Speed, km/h	Strides, n	Speed, km/h
					Lesion type			Lesion site												
					PWM	CDGM	M	ANT	POST	not def										
CP1	DI		M	1.8	1	0	0	0	0	1	26	800	15	1	60	3/3	–		19	1.2
CP2	DI		M	1.8	1	0	0	0	1	1	26	1145	20	1	41	2/2	–		20	2.3
CP3	HE	R	F	2.4	1	0	0	0	1	0	37	2630	25	1	80	0/3	–		30	2.2
CP4	DI		M	2.5	1	0	0	0	1	0	32	2000	29	2	85	2/2	–		29	1.5
CP5	DI		F	3.3	1	0	0	0	1	0	29	1760	26	1	84	3/3	–		48	2.5
CP6	DI		F	3.3	0	1	0	0	1	0	34	2065	33	1	83	2/2	–		34	1.8
CP7	DI		F	4.2	0	1	0	0	1	0	27	1010	36	2	59	3/2	–		20	2.2
CP8	DI		M	4.3	1	0	0	0	1	0	40	2670	29	2	88	2/2	–		32	2.0
CP9	DI		M	4.8	1	0	0	0	1	0	30	1570	40	2	76	3/4	–		25	2.0
CP10	DI		M	3	1	0	0	1	1	0	29	1650	20	1	80	3/3	32	1.2	72	1.9
CP11	DI		M	3.2	1	0	0	0	0	1	31	1704	19	1	87	1/2	46	1.1	78	3.0
CP12	DI		M	3.2	1	0	0	0	0	1	31	1900	20	1	76	1/2	24	1.2	58	2.8
CP13	DI		M	3.8	1	0	0	0	0	1	31	1400	15	1	95	1/2	36	1.4	76	2.5
CP14	DI		M	4	1	0	0	0	0	1	30	1450	26	1	63	2/2	30	1.3	58	2.2
CP15	DI		M	4.5	1	0	0	1	1	0	32	2000	29	2	66	2/2	48	0.7	36	0.9
CP16	DI		F	5.4	1	0	0	0	1	1	38	3110	32	2	80	3/3	32	1.1	78	2.3
CP17	DI		F	6.1	0	1	0	0	1	0	27	1500	30	1	80	2/2	42	0.9	76	3.2
CP18	DI		F	8.8	1	0	0	0	0	0	33	2500	30	1	87	2/2	30	1.3	62	3.4
CP19	HE	R	F	2.3	1	0	0	0	0	1	38	2695	18	1	61	0/1	68	1.3	74	1.9
CP20	HE	L	F	2.7	0	0	1	0	0	0	29	1215	15	1	80	1/1	20	1.7	60	2.9
CP21	HE	R	M	4	0	0	1	0	1	0	36	2650	23	1	68	0/3	66	1.1	62	3.5
CP22	HE	L	M	4.3	1	0	0	0	0	0	30	1520	26	2	46	2/2	24	2.1	64	3.7
CP23	HE	L	M	4.9	0	0	1	0	0	1	38	2200	18	1	97	3/0	82	1.3	82	2.5
CP24	HE	L	M	5.5	0	0	0	0	0	1	39	3200	16	1	79	4/4	32	2.4	76	3.6
CP25	HE	L	M	7.8	0	0	1	1	0	0	38	2820	12	1	89	4/4	26	2.0	26	2.6
CP26	HE	R	F	7.9	0	0	1	0	1	1	32	2120	25	2	98	0/1	80	1.9	36	2.5
CP27	HE	R	F	9.9	0	0	1	0	0	0	31	1200	30	2	90	4/4	16	1.8	62	4.0

*TCP* type of CP, *DI* diplegia, *HE* hemiplegia; side, affected side, *PWM* Periventricular White Matter lesions, *CDGM* Cortical and Deep Grey Matter lesions, *M* Miscellaneous (white and grey matter lesions), *ANT* Anterior lesions, *POST* Posterior lesions, *not def* not defined, *GMFC-s* Gross Motor Function Classification System, *GMFM* Gross Motor Function Measure, *MAS* Modified Ashworth Scale (the rater graded each ankle plantarflexion spasticity), *GA* gestational age at birth, *BW* birth weight, *WO* walking onset, *SW* sideways walking, *FW* forward walking. CP1–CP9: children with CP (both DI and HE) who failed to perform the SW task; CP10–CP27: DI and HE children who successfully performed the sideways task (followed by age within each group).

**Table 3:** Characteristics of typically developing (TD) children, the number of analyzed strides, and walking speeds during sideways and forward walking.

Subjects	Age, years	Gender	GA, week	BW, g	WO, month	SW		FW	
						Strides, n	Speed, km/h	Strides, n	Speed, km/h
TD1	2.4	M	40	3530	12	82	1.1	68	2.2
TD2	3	M	39	3800	15	26	1.6	72	2.1
TD3	3.8	F	38	3000	10	68	1.7	54	2.8
TD4	4.6	F	40	3000	12	34	1.3	78	2.4
TD5	5.3	F	38	2900	16	20	1.6	60	2.9
TD6	5.7	F	41	2750	15	20	1.2	70	3.9
TD7	5.8	F	38	3100	12	24	1.8	58	3.7
TD8	5.8	M	39	3100	12	26	1.7	62	3.2
TD9	6.1	F	30	1500	11	20	1.8	54	3.1
TD10	6.4	F	40	3800	12	26	1.4	60	3.5
TD11	6.8	M	38	3000	12	26	1.7	72	3.2
TD12	7.1	M	39	2900	12	32	1.9	54	3.4
TD13	7.2	M	38	2700	12	36	1.6	52	3.5
TD14	7.6	F	38	3100	12	24	2.2	58	3.3
TD15	8.8	F	38	3100	15	18	1.7	58	3.7
TD16	10.3	F	39	3900	12	24	1.9	14	2.8
TD17	10.8	M	39	3000	12	26	1.7	56	3.1
TD18	11.8	F	38	3100	12	32	1.9	44	3.9

*GA* gestational age at birth, *BW* birth weight, *WO* walking onset, *SW* sideways walking, *FW* forward walking.

All children with CP were classified between levels I and II of the Gross Motor Function Classification System (GMFCS)^31,32^. Inclusion criteria were: GMFCS < 3 and Communication Function Classification System (CFCS)^33^ ≤ 3. Exclusion criteria were lower extremity orthopaedic surgery within the past year or botulinum toxin A injections within the past 4 months. Furthermore, to assess motor function for each child with CP, we used the Gross Motor Function Measure (GMFM), a standardized observational instrument of 88 items grouped into 5 domains, related primarily to postural and locomotor abilities, with a scale of 0–100^34,35^ (Table 2). Ankle plantarflexor muscle spasticity was evaluated by the Modified Ashworth Scale (MAS)^36^ (Table 2). The assessments of GMFCS, GMFM and MAS were carried out by experienced physiotherapists in accordance with the manuals available for these instruments. All participants were able to understand the instructions and walk in an autonomous manner for the duration of the experiment. If children with CP used an ankle–foot orthosis for daily activities, it was removed for the duration of the experiment.

### Outcome measures

The primary outcome was the success or failure to perform SW locomotion. While all children were able to walk forward, some children with CP failed to walk SW (see “Results”). The SW trials were considered ‘failed’ if a child turned and walked facing forward, despite the instruction to walk sideways (the experimenter encouraged the child 3–5 times), i.e., if a child immediately rotated his/her trunk and feet by ~ 90° in the direction of progression and thus performed FW. Accordingly, we computed the percentage of subjects in each group (TD, HE and DI) with successful and failed task performance. Those subjects who failed the SW task in all (4–5) trials were excluded from all further analyses.

For the successful trials, secondary outcome measures included:

- general gait parameters (walking speed, stride length, cadence, relative stance/swing duration),
- kinematic parameters (vertical hip displacements, foot trajectory),
- range of angular motion (ROM) and muscle moments of force in the sagittal plane,
- foot placements,
- trunk yaw orientation,
- spinal locomotor output associated with muscle activity of individual legs and muscle modules.

The first two of these measures (general gait and kinematic parameters) characterize general gait performance, while the other outcome measures (ROM, muscle torques, foot placements, trunk yaw orientation, muscle activity and basic activation patterns) are specifically important to characterize interlimb coordination during movements in the frontal plane and the adopted strategy used to perform sideway stepping. The detailed description of these outcome measures is provided below.

#### General gait and kinematic parameters

The gait cycle was defined as the time between two successive foot–floor contacts by the same leg, according to the local minima of the vertical displacement of the heel (or 5MT in case of toe-walking) marker, while the local minima of the limb (GT-5MT virtual segment) elevation angle were used to define the lift-off (when the 5MT marker was elevated by > 2 cm)^37^. These criteria had been previously verified in a study on CP from the ground reaction force recordings^38^. The body was modelled as an interconnected chain of rigid segments: GH–GT for the trunk, GH–Elb for the arm, Elb–Wri for the forearm, GT–LE for the thigh, LE–LM for the shank, and LM–5MT for the foot.

The steps related to gait initiation and termination were discarded, and only those performed in the central section of the path at about constant speed were included in the analysis (2–4 strides in each trial). For SW, we characterized first the general performance, excluding children that failed to perform the task properly, and only successful trials of the participants were selected for the analysis. The number of strides during FW and SW (summed up for the trailing and leading limbs) for each child is reported in Table 2 for children with CP and in Table 3 for TD children.

Walking speed for each stride was computed as the mean speed of the horizontal trunk movement, the latter being identified by the time course of the displacement of a virtual marker located at the midpoint between left and right GT markers. Stride length was measured according to horizontal displacement of the foot marker (5MT). The vertical hip displacements (GT_z_,) was averaged across right and left legs. Foot trajectory data for HE children was presented separately for the least affected (LA) and most affected (MA) limbs. Data were time-interpolated over individual gait cycles to fit a normalized 200-point time base. The stride length, vertical hip displacements and foot trajectory were normalized to the limb length (L, determined by summing lengths of the thigh and shank segments) of the participants.

#### Additional kinematic and kinetic measurements during SW

For the SW task, in addition to the characteristics of body and foot movements in the direction of progression (frontal plane), we also characterized the limb segment (thigh, shank, foot) and joint (hip, knee, ankle) angular movements (range of angular motion, ROM) in the plane orthogonal to the direction of progression (sagittal plane) since many children with CP tended to flex the hip during swing (see “Results”). For HE children, the data are shown for both least affected (LA) and most affected (MA) limbs.

To further characterize these attempts in the sagittal plane, we calculated sagittal muscle moments about hip, knee, and ankle joints during swing phase (from toe-off to touchdown) using a Newton–Euler inverse dynamics approach^39^. Anthropometric characteristics of the thigh, shank, and foot segments in each child were estimated using values indicated by Dempster^40^. Time-varying muscle moments were normalized by individual body mass and reported (in N m kg^−1^) for comparison across participants. The peak normalized flexor moments from each joint were identified during swing phase and used for subsequent analysis. Flexor muscle moments were indicated with positive values and extensor moments with negative values.

#### Foot placements during SW

For the SW task, we also analysed foot placements of the trailing limb with respect to the leading limb. The data for right and left SW were pooled together (after an appropriate ‘flipping’ of the left stepping data in such a way that it corresponds to stepping to the right). To this end, we used the 5MT marker of the leading limb as a reference point and calculated the x,y position of the 5MT marker of the trailing limb (5MTtr_x and 5MTtr_y parameters, respectively). We categorized the percent of steps of all children with 5MTtr_x < 0 (‘normal’ SW) and 5MTtr_x > 0 (crossing one foot over the other).

#### Trunk yaw orientation during SW

The trunk yaw angle was measured as the angle between the line formed by two markers positioned on the right and left greater trochanter and the direction of SW progression. As in the case of foot placements, the data for right and left SW were pooled together (after an appropriate ‘flipping’ of the left stepping data). We computed the trunk yaw angle at the moment of the trailing limb touchdown and we analysed this angle as a function of the x-position of the trailing limb foot (5MTtr_x). Steps were categorized according to the amount of trunk rotation: with trunk yaw < 30° (relatively small trunk rotations) and with trunk yaw > 30° (noticeable trunk rotations). In the absence of trunk rotation, this angle should be close to zero.

#### EMG activity and basic activation patterns

The EMG signals were high-pass filtered (30 Hz), rectified and low-pass filtered with a zero-lag fourth-order Butterworth filter (10 Hz). The time scale was normalized by interpolating individual gait cycles of each lower limb over 200 points. We analyzed both individual muscle EMG characteristics and basic activation patterns (shared regularities across muscle activities). To highlight the usually high muscular activity of all recorded muscles during both FW and SW, we used non-normalized (in μV) EMG patterns and averaged individual muscle activity across subjects. For basic activation patterns, the EMG signals from each muscle for each leg were normalized to the peak value for each trial and each participant. Basic activation patterns were extracted from the EMG envelopes using a non-negative matrix factorization (NNMF) algorithm, as described previously^9,10,38,41^.

Briefly, NNMF was applied to all strides (for each condition) of each child to represent the $EMG$ patterns ($m\times t$ matrix) as a linear composition of basic activation patterns $P(t)$ ($n\times t$ matrix):

1 $$EMG= {\sum }_{i}^{n}{P}_{i}{W}_{i}+error, n\le m,$$

where $m$ is the number of muscles, $n$ is a predetermined number of basic patterns and $W$ ($m\times n$ matrix) correspond to weighting coefficients or muscle synergies. The $W$ and $P$ matrices are estimated to minimize the root-mean-squared error between the original $EMG$ signals and those reconstructed using $P\times W$.

Pattern decomposition was evaluated by calculating the percent of variance accounted for^42^:

2 $$VAF=sum\,of\,squared\,errors\,/\,total\,sum\,of\,squares,$$

where the total sum of squares is taken with respect to the mean over the rows of the data matrix. To determine the minimum number of basic activity patterns $n$ which best accounts for the EMG data variance, we varied the number of basic patterns from 1 to 10, and selected the smallest $n$ such that that they accounted for ≥ 80% of the variance of EMG profiles when using Eq. (1).

The structure of muscle modules was compared using a cluster analysis. The number of clusters corresponded to the number of similar muscle synergies across participants and conditions. In order to compare our results with previous data^9,38,43–45^, and consistent with the NNMF results (see “Results”), we set the number of clusters to 4. To match similar modules across subjects and conditions, all muscle synergies (W, Eq. 1) extracted in each subject from trailing and leading limbs during SW and from the right limb during FW were pooled together and partitioned in 4 mutually exclusive clusters using the k-means algorithm^46^. For each cluster and each subject, we evaluated the similarity of the average basic activation patterns (P, Eq. 1) obtained for FW with the average patterns from the same cluster for trailing and leading leg in SW using the cosine of the angle α between the patterns.

To characterize differences in the timing of EMG bursts and basic activation patterns between groups, we computed the center of activity $(CoA$) during the gait cycle^9,10,38^. These parameters were calculated over individual strides and averaged.

The CoA was calculated using circular statistics^47^ as the angle of the vector (1st trigonometric moment) in polar coordinates (polar direction denoted the phase of the gait cycle, with angle θ that varies from 0 to 360°) that points to the center of mass of that circular distribution using the following equations:

3 $$A=\sum_{t=1}^{200}\left(\mathrm{cos}{\theta }_{t}\times {P}_{t}\right),$$

4 $$B=\sum_{t=1}^{200}\left(\mathrm{sin}{\theta }_{t}\times {P}_{t}\right),$$

5 $$CoA={\mathrm{tan}}^{-1}\left(B/A\right).$$

The $CoA$ provides an estimate of the timing of the EMG bursts. It was chosen because it was impractical to reliably identify a single peak of activity in the majority of muscles. In some cases, averaging between distinct foci of activity may result in a poorly representative CoA in the intermediate zone. However, CoA generally helps understanding if the distribution of muscular activity remains unaltered^41^ across different groups of children and muscles during forward and sideways walking.

### Statistics

Descriptive statistics included the calculation of the mean and standard deviation (SD) of the assessed variables. Parametric statistics were used after determining that the data were normally distributed (Kolmogorov–Smirnov test, p > 0.2 for all variables). Repeated-measures (RM) ANOVA was used to evaluate the effect of group (between factor), walking condition (within factor) and the interaction of the two factors on the walking speed. One-way ANOVA was used to evaluate differences on the joint peak moments and GT_z_ trajectory between groups. Tukey honestly significant difference (HSD) post hoc test was used to compare means. Statistics on correlation coefficients was performed on the normally distributed, Z-transformed values. Statistical analysis of circular data^47^ was used to characterize the $\mathrm{CoA}$ and its variability across steps. In particular, the Harrison-Kanji test was used to test the influence of group and body side, while the Watson-Williams test was used for the post-hoc comparison. We used the Rayleigh test for non-uniformity of circular data to check whether CoA samples were distributed uniformly around the cycle or had a common mean direction. Reported results are considered significant for p < 0.05.

## Results

### Participants’ characteristics

Twenty-seven children with CP and eighteen age-matched TD children participated in the study. Ten children with a clinical diagnosis of hemiplegia (HE) due to CP (age range 2.3–9.9 years; 5 right and 5 left hemiplegia) and seventeen children with diplegic (DI) CP (age range 1.8–8.8 years) were recruited. General and individual CP participant’s’ characteristics are presented in Tables 1 and 2, respectively. TD children, born at term, clinically defined as typically developing by their paediatrician (age range 2.4–11.8 years; individual characteristics are listed in Table 3) were also recruited.

### Task performance and general gait parameters

**Figure 1:**
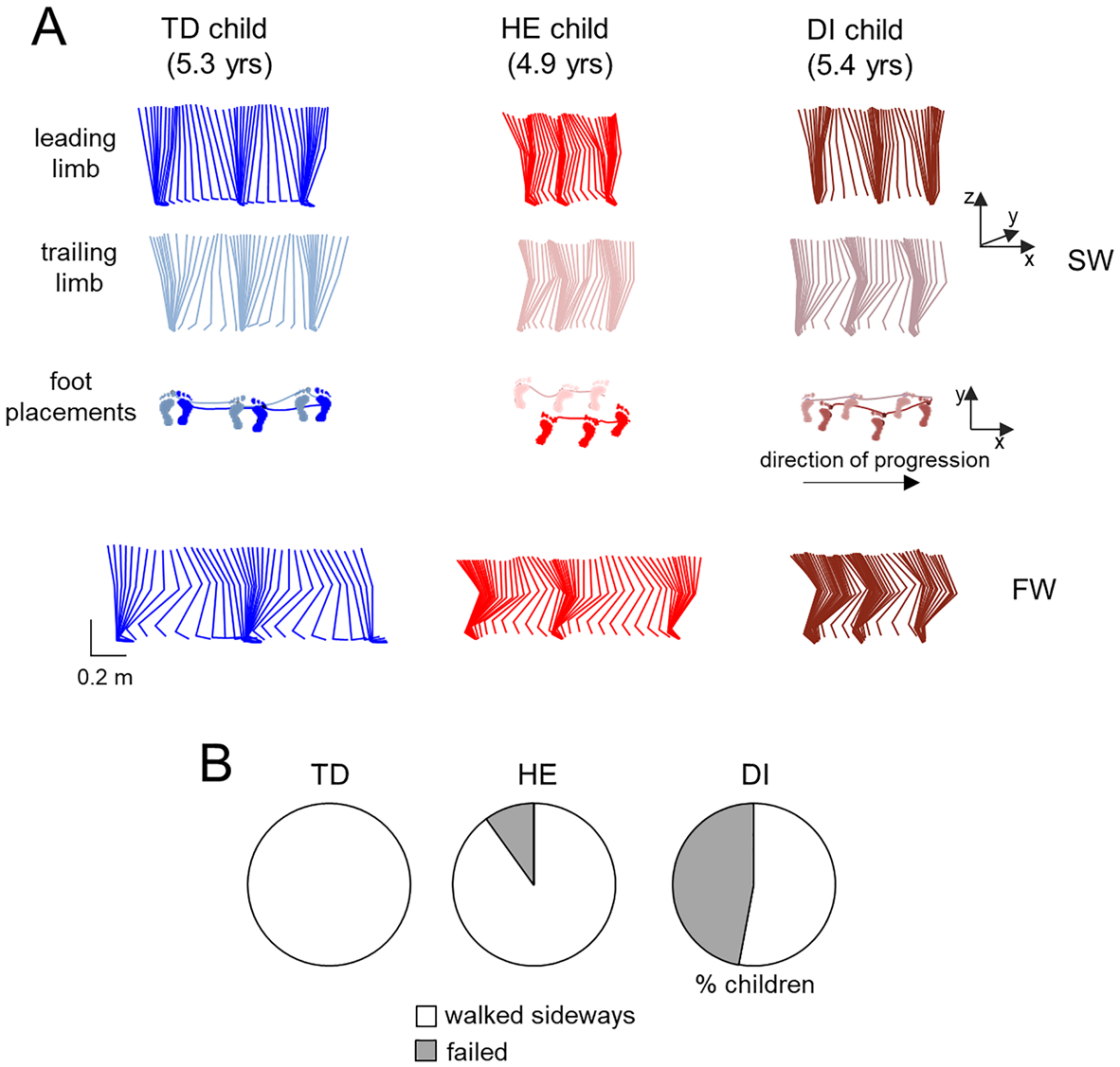
General SW task performance. (**A**) Examples of unilateral stick diagrams during two consecutive strides with SW (upper panels) and FW (lower panels) task performance in a typically developing (TD) child (5.3 years), hemiplegic (HE) child (4.9 years) and diplegic (DI) child (5.4 years). For the SW task, stick diagrams of both leading and trailing limbs are shown along with corresponding foot placements and foot (VM marker) trajectories in the horizontal plane. (**B**) Pie charts showing the percentage of children for each group with successful or failed performance of SW.

Figure 1A shows examples of leading and trailing limb movements (stick diagrams) and foot placements during SW (upper panels) and corresponding performance of FW (lower panels) in one TD child, one HE child, and one DI child. All TD children successfully performed the SW task (Fig. 1B left panel) although they typically walked slower and with shorter steps than during FW (Fig. 1A). Instead, children with CP showed difficulties when performing the SW task. Figure 1B illustrates the percentage of subjects in each group (TD, HE and DI) with successful and failed task performance, while Table 2 reports the characteristics of children with CP who failed to perform the SW task (CP1–CP9). In particular, only 18 out of 27 children with CP were successful in performing SW. Remaining 9 children (8 DI children and 1 HE child, aged 1.8–4.8 years, Table 2) turned and walked facing forward, i.e. they tended to rotate their trunk and turn in the direction of progression, so that they actually walked forward despite the fact that the experimenter showed them (both to CP and TD children) how to step sideways. They had mostly posterior lesions of the brain and/or periventricular leukomalacia (Table 2). This tendency to walk forward instead of SW was observed in all (4–5) trials of ‘SW’ that these children tried to execute. These subjects were excluded from all further analyses. Nevertheless, it is worth stressing that, in spite of the failure to step sideways, they successfully performed the normal FW task without any arm support (Table 2).

**Figure 2:**
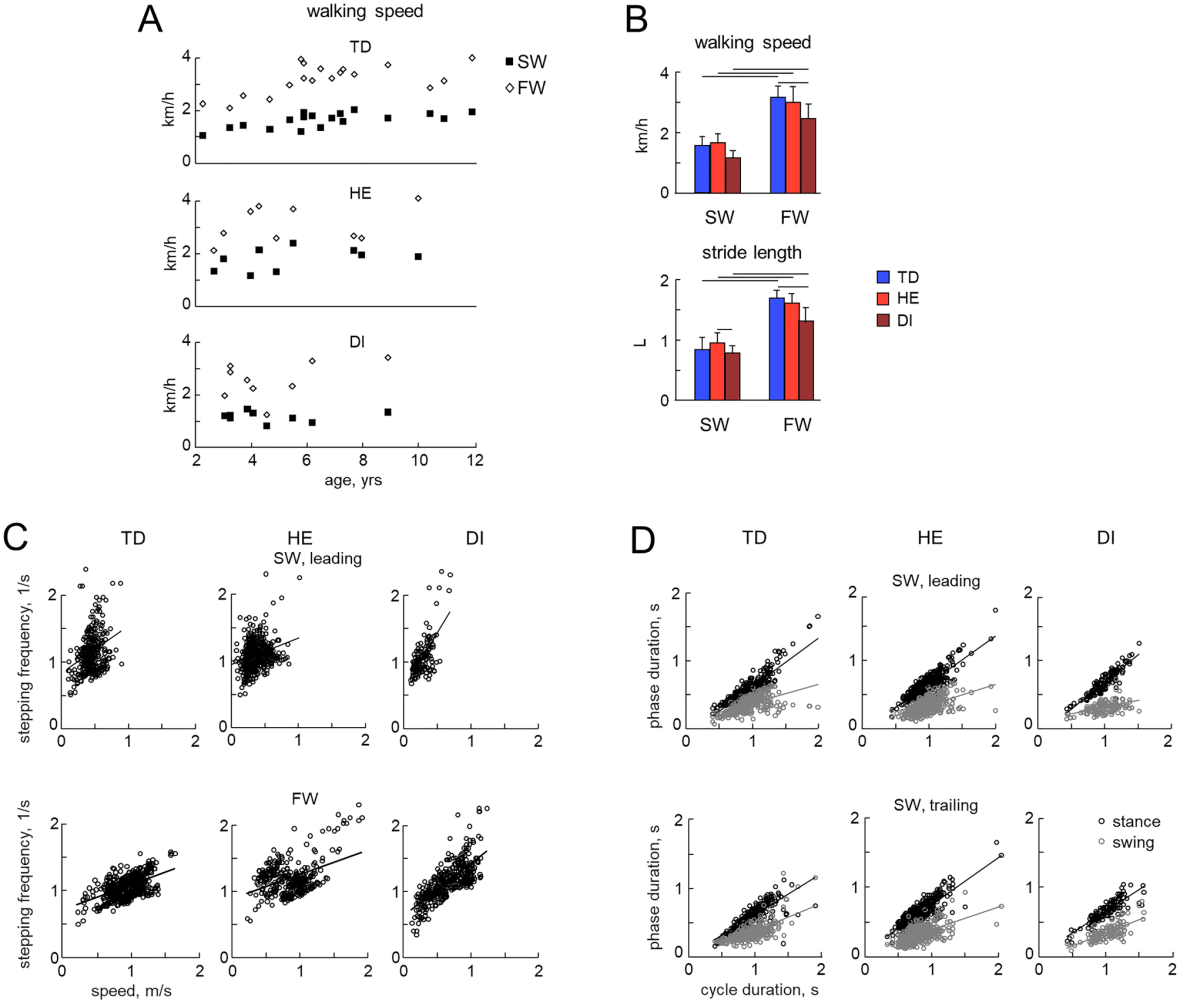
General gait parameters during sideways and forward walking in children with CP and TD children. (**A**) Walking speeds in TD, HE and DI children (each point corresponds to the mean speed in 1 individual child). Data are plotted as a function of age. (**B**) Speed and stride length for SW (for both leading and trailing limbs) and FW. Stride length was normalized to the limb length L (thigh + shank). Horizontal lines denote significant differences (RM ANOVA with Tukey’s HSD, p < 0.05). (**C**) Stepping frequency vs. speed during SW (leading leg) and FW (data for right and left legs were pooled together). Each data point represents one stride. (**D**) Phase duration vs. cycle duration for the trailing and leading limbs during SW (black circles—stance, and grey circles—swing phase). Solid lines represent linear regressions.

**Figure 3:**
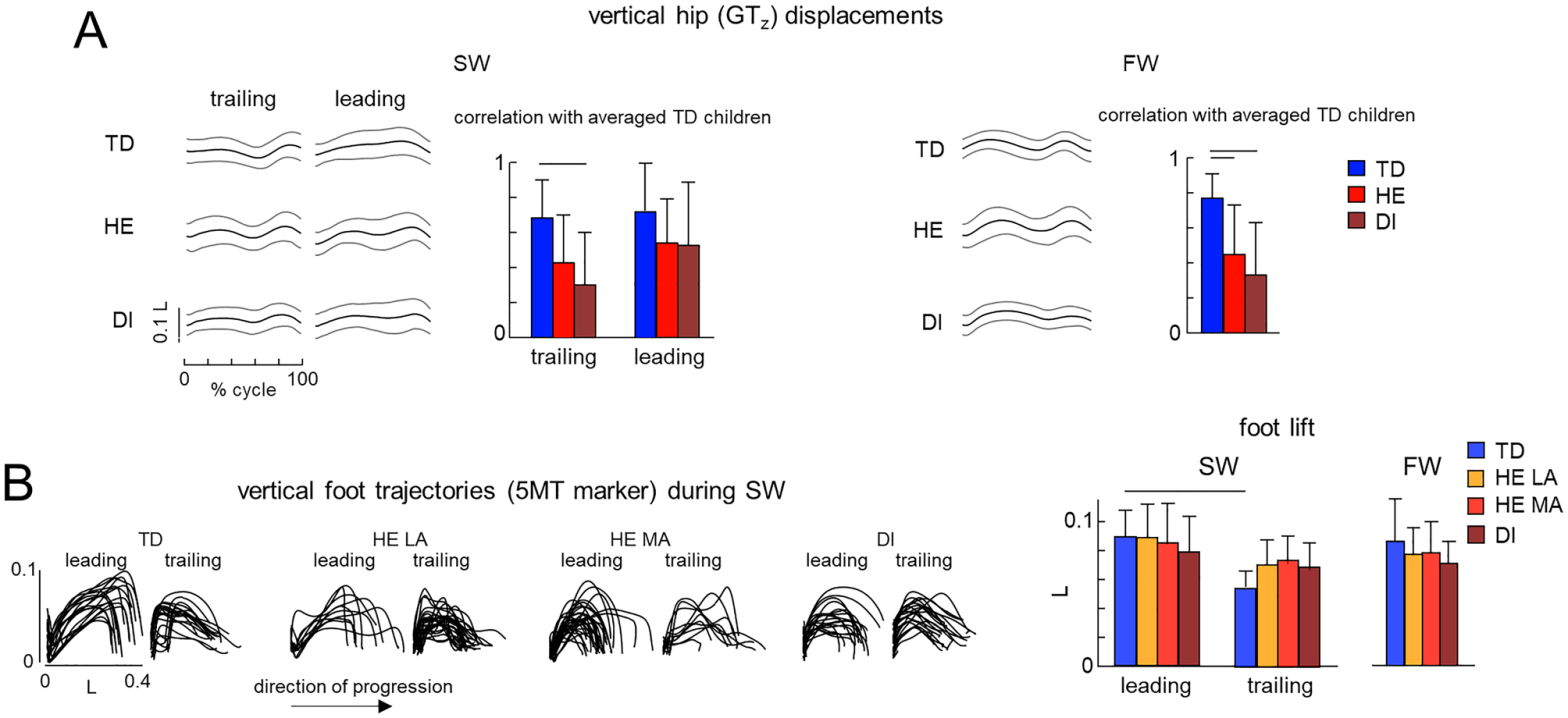
Vertical hip joint and foot displacements during SW and FW. (**A**) Time course of vertical hip displacements (GTz, normalized by the limb length L and averaged across right and left legs) averaged across subjects (mean ± SD, left panels) and correlation with the ensemble-averaged data for TD children (right panel). Horizontal lines denote significant differences (one-way ANOVA with Tukey’s HSD, p < 0.05). (**B**) Left: examples of superimposed foot (5MT marker) trajectories of the leading and trailing limbs during SW in 1 TD child (7.2 years), 1 HE child (4.9 years) and 1 DI child (6.1 years); right: vertical foot (5MT) excursion (mean + SD) of the leading and trailing limbs for sideways and forward walking, expressed in relative units. Horizontal line denotes significant differences between limbs (RM ANOVA with Tukey’s HSD, p < 0.05). The data for HE children was presented separately for the least affected (LA) and most affected (MA) limbs.

General gait parameters in children who succeeded SW are illustrated in Figs. 2 and 3. It is also worth noting that HE children who succeeded SW in the direction of the most affected (MA) limb, performed SW in the direction of the least affected (LA) limb as well. During FW and SW trials, participants performed several steps at approximately constant speed, which increased with age (Fig. 2A, left panels).Nevertheless, all children (TD and CP) performed SW at a slower speed than during FW (RM ANOVA, effect of walking condition F(1,33) = 167.3, p < 0.001, Tukey HSD p < 0.001 for TD, DI and HE, Fig. 2B, upper panel). The preferred walking speed was also slower for DI children compared to TD children during FW (RM ANOVA, effect of group F(2,33) = 6.91, p = 0.003, Tukey HSD p = 0.02, Fig. 2B). The stride length was roughly twice as short during SW than it was during FW in all children (RM ANOVA, effect of walking condition F(1,33) = 312.6, p < 0.001, Tukey HSD p < 0.001 for all groups, Fig. 2B, lower panel). Despite some variability, the cadence increased with increasing walking speed (Fig. 2C) and the swing phase durations were shorter than the stance phase durations (Fig. 2D), in agreement with previous studies on SW in infants^48^.

Figure 3 illustrates vertical displacements of the hip and foot. During FW, the temporal profile of the vertical hip displacements (GT_z_) of TD children typically exhibited two peaks over gait cycle, coinciding with mid-stance of the right and left legs (Fig. 3A). In children with CP, GT_z_ oscillations were more variable from step to step, in accordance with previous studies^38^, and their profile showed relatively low correlations with the ensemble-averaged profile for TD children (0.45 ± 0.28 and 0.33 ± 0.23 for HE and DI children, respectively). During SW, the two-peaked GT_z_ trajectory was less evident and the correlations with the averaged profile of TD children were lower for HE and DI children (Fig. 3A left panels). Children lifted the foot to about the same extent during FW and SW, although the foot elevation was significantly higher for the leading limb with respect to the trailing limb in TD children (RM ANOVA, effect of limb F(1,41) = 39. 7, p < 0.001, Tukey HSD, p < 0.001), while it did not differ for children with CP (Tukey HSD, p > 0.36 for all comparisons) (Fig. 3B).

### Angular movements and joint muscle moments in the sagittal plane during SW

**Figure 4:**
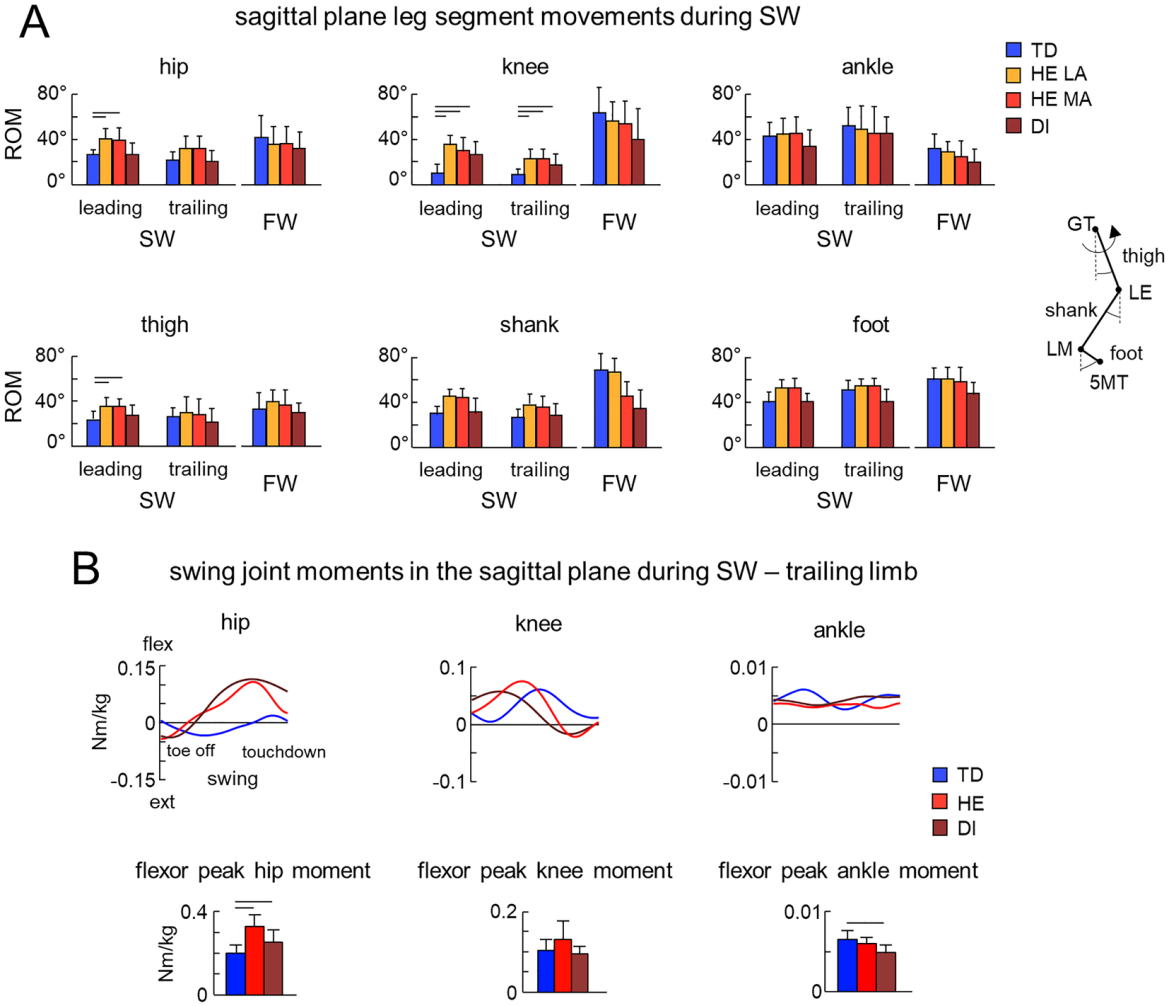
Angular movements and joint moments in the sagittal plane during SW. (**A**) Ranges of angular flexion–extension motion (ROM, mean + SD) of joint angles (hip, knee, ankle) and elevation angles (thigh, shank, foot). Horizontal lines denote significant differences (RM ANOVA with Tukey’s HSD, p < 0.05). (**B**) Top: trailing limb muscle moments (normalized by body weight) about the hip, knee, and ankle joints in the sagittal plane from representative participants (TD, 11.8 years; HE, 7.9 years; DI, 8.8 years) during swing of the leg (from toe-off to touchdown). Bottom: group averaged (+ SD) bar graphs for trailing limb flexor peak hip, knee, and ankle muscle moments during swing. Horizontal lines denote significant differences (one-way ANOVA with Tukey’s HSD, p < 0.05).

The kinematic analysis of SW in children with CP revealed the notable frequent presence of ‘elements’ of movements in the sagittal plane (i.e., in the plane orthogonal to the direction of progression, flexion—extension motion) while the sideways movement was primarily performed laterally in the frontal plane. For instance, in the illustrations presented in Fig. 1A, note somewhat flexed ‘knee’ (projected on the plane of progression) joint in HE and DI children during the swing phase of SW, reflecting attempts to produce forward (sagittal plane) kinematics. The HE child (middle column) also tended to rotate the trunk and cross one leg over the other toward the direction of SW progression (see foot placements of the trailing limb). We specifically analyzed these movements of the leading and trailing limbs and summarized the results in Fig. 4.

During SW, some leg joint flexion in the sagittal plane is necessary to raise the leading and trailing legs to avoid stumbling and provide an appropriate foot trajectory during the swing phase. Nevertheless, these angular joint movements in the sagittal plane are relatively small. Indeed, in TD children, the ROM of the proximal hip and knee joint angles (~ 22° and ~ 10°, respectively, Fig. 4A upper panels) was notably smaller than during FW. Only the ROM of the distal ankle joint (~ 45°) was larger, likely to provide the appropriate foot-strike and push-off phases of SW. The ROMs of the limb segment elevation angles followed a similar trend in TD children (Fig. 4A lower panels).

In children with CP, we found significantly larger angular movements in the proximal joints during SW with respect to TD children (Fig. 4A), even though the walking speed was similar or even slower in children with CP. In particular, for the leading limb, the ROM was larger for the hip joint in HE LA and HE MA (RM ANOVA, effect of group F(3,41) = 11.4, p < 0.001, Tukey HSD p < 0.009 for all comparisons) and for the knee joint in HE LA, HE MA and DI (RM ANOVA, effect of group F(3,41) = 29.4, p < 0.001, Tukey HSD p < 0.001 for all comparisons) with respect to TD children. For the trailing limb, the ROM war also larger for the knee joint in HE LA, HE MA and DI (RM ANOVA, effect of group F(3,41) = 29.4, p < 0.001, Tukey HSD p < 0.006 for all comparisons). The ROMs of the limb segment elevation angles showed similar differences between CP and TD children (Fig. 4A lower panels). The ROM of the distal ankle joint was similar in CP and TD children. During SW in HE children, there were similar ROMs of the LA and MA limbs when they played the leading or trailing role (Fig. 4), as well as similar foot lifts (Fig. 3B right panel).

We also analyzed the joint muscle moments during elevation of the foot in the sagittal plane (orthogonal to the direction of progression) to examine the redistribution of muscle torques across the leg joints in children with CP with respect to TD children. Representative sagittal joint muscle moments of the trailing limb are illustrated for the three (TD, HE and DI) participants in Fig. 4B (upper plots). In these plots, flexor muscle moments are indicated with positive values and extensor moments with negative values. HE and DI children had significantly larger hip muscle flexor moments (Fig. 4B), which is consistent with the findings presented below and suggests a propensity to walk forward. In fact, a main effect across groups was observed in the peak hip flexor moment (one-way ANOVA, effect of group F(6,62) = 8.69, p < 0.001, Tukey HSD, p < 0.02, Fig. 4B lower plots). No significant difference in the knee moment was found, however, another effect of group was observed in the peak ankle flexor moment, and post hoc analysis indicated its decrease in children with DI (one-way ANOVA, effect of group F(6,62) = 8.69, p < 0.001, Tukey HSD, p < 0.002).

### Foot placements and trunk orientation during SW

**Figure 5:**
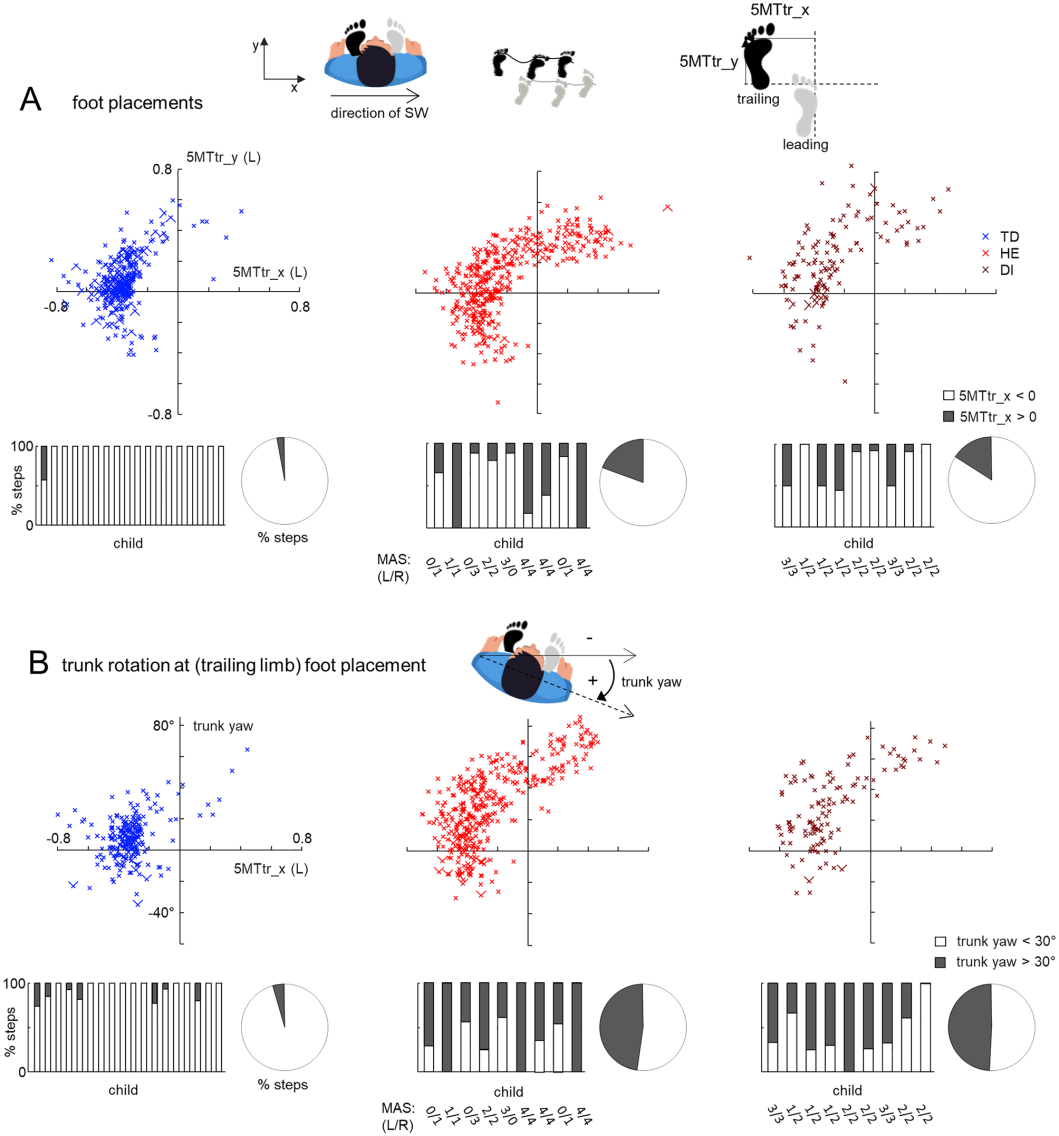
Foot placements and trunk yaw orientation during SW in TD, HE and DI children. (**A**) Upper panels—spatial distribution of trailing limb foot placements (5MT marker) relative to the leading limb foot position (5MTtr_y vs. 5MTtr_x, frame of reference that originates on the 5MT marker of the leading leg is shown on the top) in all strides of TD, HE and DI children. Each point corresponds to individual steps. Bottom panels: percentage of steps for each child (left) and pie chart that shows the percent of steps of all children (right) with 5MTtr_x < 0 (‘normal’ SW) and 5MTtr_x > 0 (crossing one foot over the other). For children with CP, we also indicated the spasticity (MAS) scores. (**B**) Trunk (pelvis) yaw orientation at trailing limb foot touchdown as a function of the x-position of the trailing limb foot (5MTtr_x). A similar format as in panel (**A**). Steps on the bottom were categorized according to the amount of trunk rotation: with trunk yaw < 30° and with trunk yaw > 30°. Children on the bottom plots are ordered (from left to right) according to their age. While the frames of reference in panels (**A**) and (**B**) correspond to sideways stepping to the right, the data for right and left SW were pooled together (after an appropriate ‘flipping’ of the left stepping data in such a way that it corresponds to stepping to the right).

In addition to the elements of ‘forward’ (hip joint angle) movements during SW in children with CP (Fig. 4), we also found the frequent attempts to slightly turn their body in the direction of body progression (Fig. 5). Figure 5 illustrates the percentage of foot placements with crossing one foot over the other (A) and with noticeable trunk yaw rotations (B), which can be interpreted as the attempts to turn in the direction of body progression.

In TD children, the percent of such steps was relatively small. For instance, only one TD child demonstrated some steps with crossing one foot over the other (Fig. 5A, left) and he was the youngest one (2.4 years, Table 3). In children with CP, the portion of steps with a tendency to turn in the direction of progression was significantly larger and such step were observed in almost all children independent of age. Overall, the portion of steps with crossing one foot over the other (5MTtr_x > 0) was 3% in TD, 22% in HE and 16% in DI (pie charts in Fig. 5A), and the number of steps with notable trunk yaw rotation (trunk yaw > 30°) was 6% in TD, 47% in HE and 49% in DI (pie charts in Fig. 5B). It is also worth noting that the strides with the above-mentioned elements of turning were observed in children with CP throughout the whole experiment, i.e. during both the first trial and subsequent trials.

### EMG patterns

**Figure 6:**
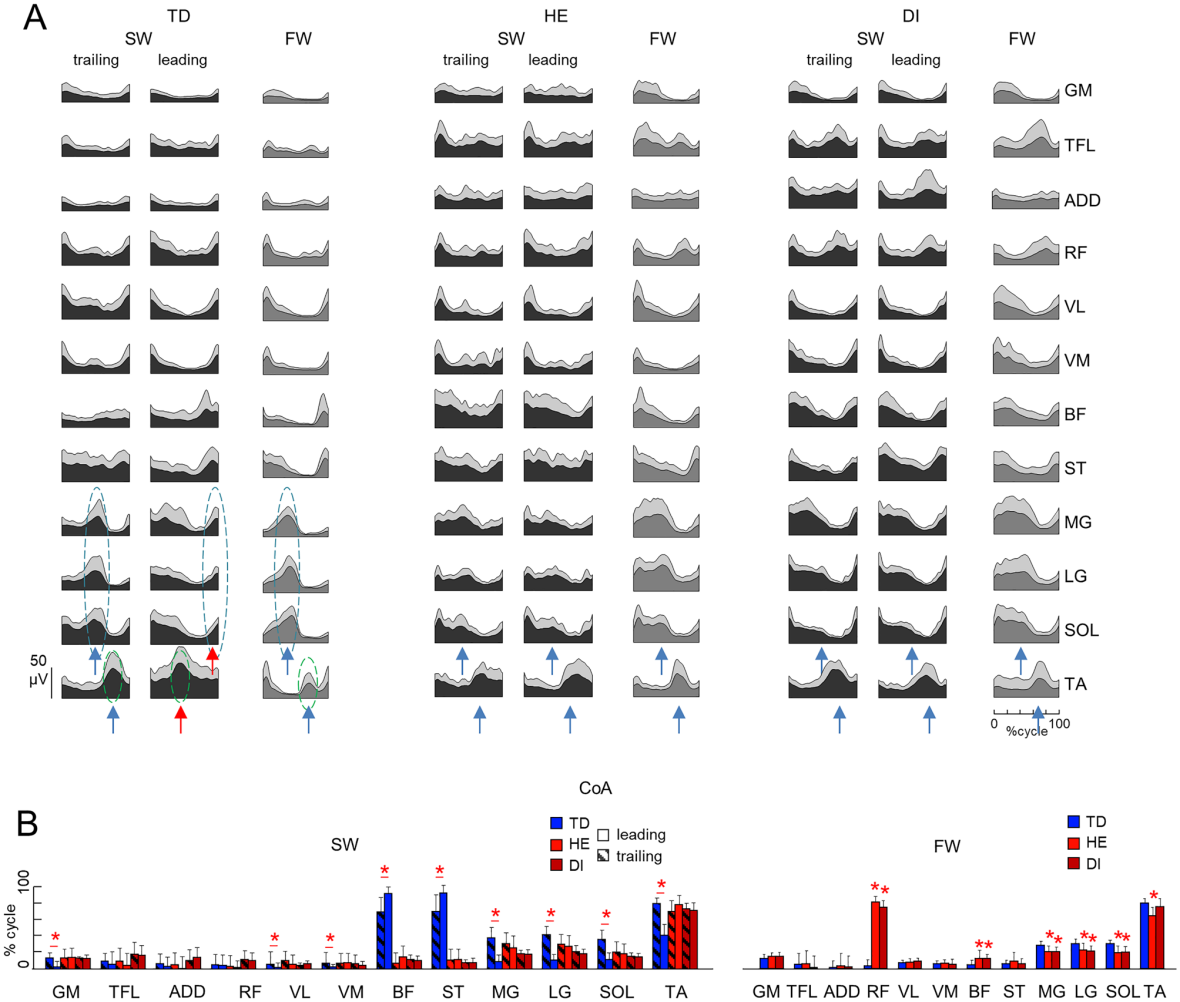
Characteristics of EMG activity. (**A**) Ensemble averaged (+ SD) EMG activity patterns of 24 bilateral leg muscles recorded in TD, HE and DI children. For SW, EMG activity is shown for both leading and trailing limbs. EMG data are plotted versus normalized gait cycle. (**B**) Center of activity (CoA) (mean + SD). For SW, asterisks denote significant differences between the limbs, while for FW, asterisks indicate differences with TD children (post-hoc Watson-Williams test p < 0.05). Red arrows schematically emphasize a significant shift of the timing of activity of TA and calf muscles of the leading limb in TD, while blue arrows point to relatively similar activity of these muscles in SW and FW. *ADD* adductor, *GM* gluteus maximus, *LG* gastrocnemius lateralis, *MG* gastrocnemius medialis, *RF* rectus femoris, *SOL* soleus, *TA* tibialis anterior, *TFL* tensor fascia latae, *VL* vastus lateralis, *VM* vastus medialis.

The ensemble-averaged EMGs of all recorded muscles are illustrated in Fig. 6A for both trailing and leading limbs during SW and for FW. Despite inter-individual variability, the lower limb muscle activity patterns exhibited task-specific and group-specific differences.

During FW, there were some significant differences between groups. For instance, in most children with CP, there was prominent activity in the ankle extensors (SOL, LG, MG) throughout the entire stance phase, whereas in TD children the activity of these muscles was typically observed in late stance (Fig. 6A, FW). As a consequence, there was a shift of the center of activity (CoA, see “Methods”) of these muscles toward early stance (Fig. 6B). The activity of TA showed only one major peak at the onset of swing in children with CP but two prominent peaks in TD children. In accordance with previous studies^9,10,38^ and with increased coactivation of antagonist muscles in CP^49,50^, the major bursts of activity of most muscles were wider in children with CP with respect to TD children during FW.

During SW, there was a substantial difference in the muscle activity of the trailing and leading limbs in TD children, while these interlimb differences were absent or less evident in children with CP. Specifically, in TD children, the BF, ST, MG, LG, SOL and TA activity of the leading limb showed systematic changes with respect to FW and with respect to the trailing limb, so that the CoA of these muscles significantly differed between limbs (Watson-Williams post hoc test, p < 0.001; Fig. 6B). In particular, the activity of TA of the leading limb shifted towards mid-stance, while the activity of calf muscles clearly shifted towards end-swing and onset of stance (Fig. 6B, also schematically shown by red arrows in Fig. 6A). In children with CP, there was a lack of such interlimb differences and the activity of these muscles resembled that of FW (Fig. 6). In HE children, despite some variability, there were similar EMG envelopes of the LA and MA limb muscles during SW when the LA and MA limbs played the leading or trailing role, so that we pooled together the data of the LA and MA limbs in Fig. 6. In sum, in TD children there was a clear differentiation of the lift-off related muscle activity between the leading and trailing limbs during lateral sidestepping, whereas there was a lack of adjustments in children with CP, who strikingly exhibited similar muscle patterns for FW and SW.

### Basic muscle activation patterns and muscle synergies

**Figure 7:**
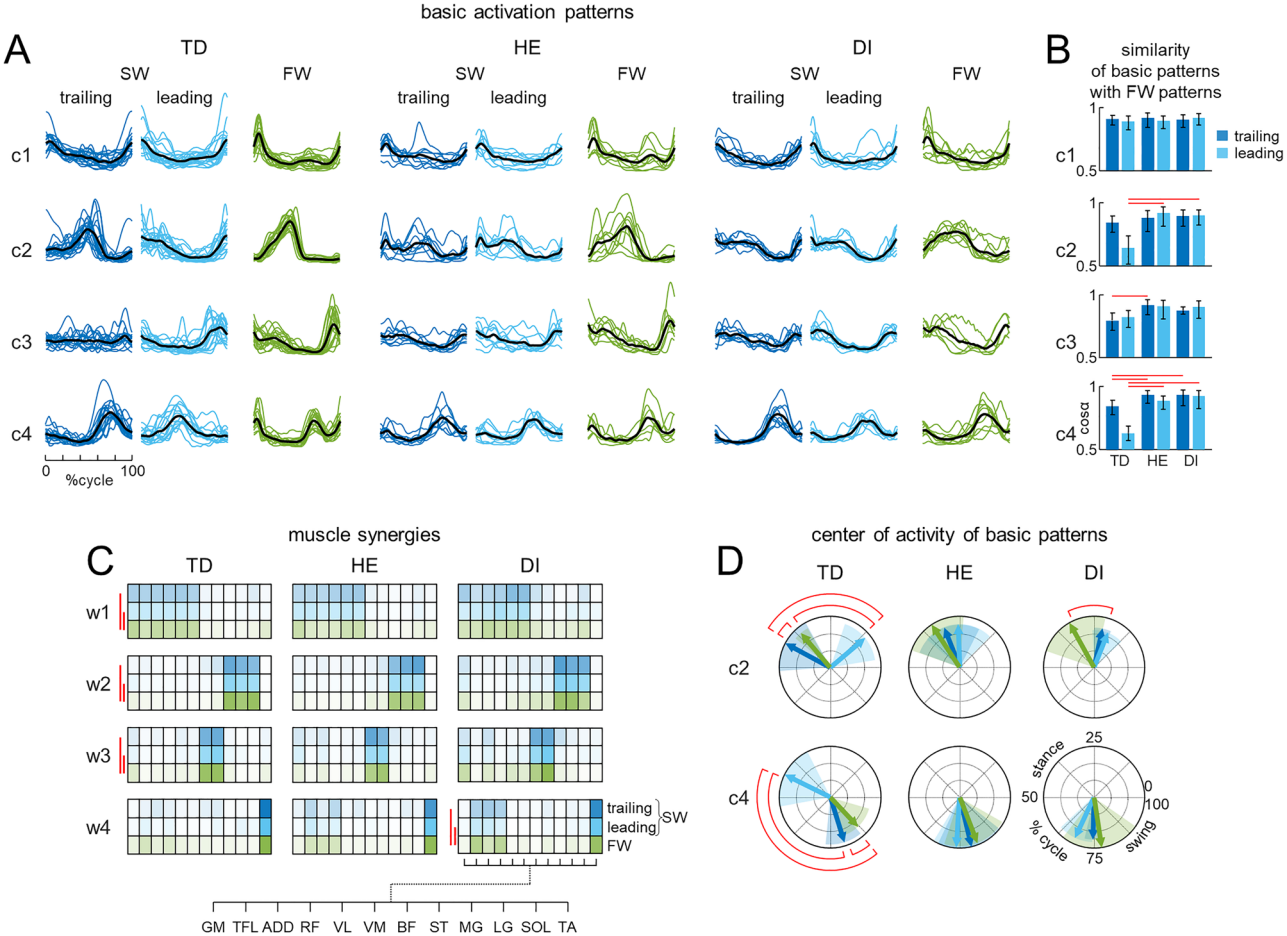
Statistical analysis of EMG patterns during SW and FW using non-negative matrix factorization. (**A**) Basic activation patterns in TD children and in children with HE and DI. Each curve represents the mean (across strides) pattern for an individual child, average patterns across children are illustrated with black lines. Data for the trailing (dark blue lines) and leading (light blue lines) limb are shown separately for SW, data for the right leg are shown for FW (green lines). The basic patterns from the same cluster (c1–c4; based on k-means clustering on muscle synergies) are plotted in a “chronological” order (with respect to the timing of the main peak in FW). (**B**) Average (± 95%CI, z-transformed, across subjects) similarity (cosα) of the basic activation patterns (c1–c4, from top to bottom) in FW with the basic patterns in trailing and leading limbs during SW. Red lines denote significant (post-hoc Tukey–Kramer multiple comparison p < 0.05) differences between TD and HE or DI children. (**C**) Muscle synergies (weighting coefficients w1–w4) of corresponding basic patterns (c1–c4) plotted in colour scale. Each row represents a condition (leading/trailing limb for SW and right limb for FW) and each column represents a muscle, the intensity of the colour is proportional to the muscle weight. Red lines denote significant (post-hoc Tukey–Kramer multiple comparison p < 0.05) differences between conditions. (**D**) Polar plots of the centre of activity (CoA) of “adaptable” c2 and c4 activation patterns for each group. Polar direction denotes the relative time over the gait cycle (time progresses counter-clockwise), radius of the vector denotes the average (across subjects) maximum amplitude of the basic pattern and the width of the sector denotes angular SD. Red lines denote significant (circular Watson-Williams test with Bonferroni correction for multiple comparisons p < 0.05) differences across conditions.

In line with previous studies^9,38,51–54^ the dimensionality of multi-muscle EMG activity during walking was described by a small number of motor modules (Fig. 7). On average (± SD) 4.3 ± 0.8 modules were sufficient to explain at least 80% of the VAF of the EMG activity during FW (4.4 ± 0.7 for TD, 4.8 ± 0.8 for HE and 3.8 ± 0.4 for DI children), 5.4 ± 0.8 modules were sufficient to explain at least 80% of the VAF of the EMG activity of the trailing leg during SW (5.4 ± 0.8 for TD, 5.8 ± 0.4 for HE and 5 ± 0.7 for DI children), and 5.2 ± 0.85 modules were sufficient to explain at least 80% of the VAF of the EMG activity of the leading leg during SW (5.3 ± 0.8 for TD, 5.4 ± 0.5 for HE and 4.7 ± 1 for DI children). To match similar muscle modules across participants and conditions, we performed a k-mean clusterization on the muscle synergies of all subjects from all conditions (SW-trailing, SW-leading, and FW) pooled together. In order to compare our results with the previous data^9,10,38^, and consistent with the NNMF results for FW, we set the number of clusters to 4. The basic activation patterns corresponding to each cluster of synergies were averaged across strides for each participant in each condition (Fig. 7A).

In TD children, the basic patterns of the first and third cluster (c1 and c3 in Fig. 7A) were quite similar between FW and SW (average scalar product ≥ 0.89 in c1 and ≥ 0.8 in c3 for trailing and leading limb, Fig. 7B), while the basic patterns of the second and fourth cluster, which are associated with muscle synergies involving distal leg muscles (c2 and c4 and w2 and w4 in Fig. 7A,C), were more “adaptable” to SW, especially in the leading limb (average scalar product ≤ 0.64 for c2 and c4 patterns of the leading leg, Fig. 7B). These changes were reflected by a significant average shifting of the CoA from FW to SW leading leg of ~ 25% and 44% of the gait cycle, respectively for c2 and c4 patterns (Watson-Williams test, effect of condition F(2,53) = 70, p < 0.001 for c2 and F(2,45) = 77, p < 0.001 for c4 and Watson-Williams test with Bonferroni correction, p < 0.001 for both comparisons, Fig. 7D). Also, the CoA of c2 and c4 basic patterns of the trailing limb during SW resulted slightly but significantly shifted compared to FW in TD children (~ 6% and 7% of the gait cycle respectively for c2 and c4, Watson-Williams test with Bonferroni correction, p = 0.048 for both comparisons, Fig. 7D).

In children with CP, the same basic activation patterns did not show the same adjustments from FW to SW. In fact, the similarity of c2 and c4 basic activation patterns of the leading limb during SW with the corresponding patterns during FW was significantly higher in HE and DI children compared to TD children (RM-ANOVA, effect of group F(2,33) = 7.2, p = 0.003, Tukey’s HSD test, p < 0.001 for HE and p = 0.001 for DI for c2 and RM-ANOVA, effect of group F(2,24) = 16, p < 0.001, Tukey’s HSD test, p < 0.001 for both HE and DI, Fig. 7B). For c4, also the similarity of basic patterns of the trailing limb during SW with the corresponding patterns during FW was significantly higher in HE and DI children compared to TD children (Tukey’s HSD test, p = 0.04 for HE and p = 0.009 for DI, Fig. 7B). Moreover, the CoA of c2 and c4 basic patterns was not significantly shifted in HE children nor in the trailing nor in the leading limb (Watson-Williams test, effect of condition, p = 0.45 for c2 and p = 0.43 for c4, Fig. 7D) and in DI children resulted slightly (~ 14% of the gait cycle) significantly shifted only in c2 in the leading leg (Watson–Williams test, effect of condition, p = 0.009 and Watson-Williams test with Bonferroni correction, p = 0.04, Fig. 7D) but not in the trailing leg (Watson-Williams test with Bonferroni correction, p = 0.068, Fig. 7D) and not significantly shifted in c4 (Watson-Williams test, effect of condition, p = 0.15, Fig. 7D). Moreover, the muscle synergies of all clusters were significantly affected by condition in TD children (RM-ANOVA effect of condition F(2,30) = 9.5, p < 0.001 for w1, F(2,32) = 10, p < 0.001 for w2, F(2,26) = 20, p < 0.001 for w3, and F(2,20) = 5.8, p = 0.01 for w4, Fig. 7C) but not in HE children (RM-ANOVA effect of condition F(2,16) = 0.58, p = 0.57 for w1, F(2,16) = 1.3, p = 0.29 for w2, F(2,14) = 0.62, p = 0.55 for w3, and F(2,12) = 2.1, p = 0.17 for w4, Fig. 7C) and only in w4 in DI children (RM-ANOVA effect of condition F(2,16) = 2.4, p = 0.12 for w1, F(2,16) = 0.24, p = 0.24 for w2, F(2,10) = 2.6, p = 0.12 for w3, and F(2,14) = 8.1, p = 0.005 for w4, Fig. 7C).

## Discussion

We reported in detail the results of an observational cross-sectional study comparing walking in two different directions, forward (FW) and sideways (SW), between two populations of participants, children with CP and typically developing children. The results in children with CP walking FW were fully consistent with our previous results^3,9,10,38^. The novel results concern walking SW. These revealed specific developmental deficits in the performance of SW and spatiotemporal organization of left and right leg muscle activity in children with CP (Figs. 4, 5, 6 and 7).

### Cerebral dysfunction and general performance of the SW task

In general, children with CP have difficulties in performing SW. About 30% of them failed on the primary outcome measure, i.e. they were unable to perform the SW task (while the experimenter showed them how to step sideways), even though they were able to walk forward without any arm support. Strikingly, their GMFCS/GMFM scores were similar to those of children who successfully performed sideways walking (Table 2). Interestingly, most of them were diplegic and they had mostly posterior lesions of the brain, as well as periventricular leukomalacia (Table 2), possibly in relation to their deficits in the important role of periventricular white matter and parietal lobe activity in visually planning gait adaptations^55–58^ since SW may rely more on visually guided movements and foot trajectory control than FW. The available visual field during SW is constrained and may present an additional challenge for children with CP who are more reliant on visual input during walking. For example, even during FW, individuals with CP show a higher sensitivity and higher foot placement responses to visual stimuli compared to the TD group^59^. The results support the idea that directional locomotor movements, such as sidestepping (Table 2) and backward walking^9^, can be used for more comprehensive diagnosis of CP as a reliable clinical measurement tool for the assessment of advanced locomotor ability, as well as for gait assessment and rehabilitation. Impaired SW task performance may reflect developmental deficits in the adaptable control of gait, along with other difficulties in performing locomotor movements included in the GMFM assessment of persons with CP.

Children with CP, who succeeded with sidestepping (∼ 70%, Table 2), performed the task differently than age-matched TD children even though there were some similarities. As for similarities, in all children groups, the strides were nearly twice as short and the walking speed was about two times slower during SW than it was during FW (Fig. 2A,B). While the developmental trend was less noticeable in children with DI (likely due to inter-individual variability and more unstable or cautious gait), the mean walking speed increased with age during both FW and SW, in agreement with the developmental growth of the body height (Fig. 2A). However, an idiosyncratic feature of SW in children with CP was the presence of the elements of movements in the sagittal plane while moving sideways. Although the bipedal inverted-pendulum model of an ‘idealized’ sideways gait entails the sequence of hip lowering, foot-strike and push-off impulses, and subsequent hip raising in the frontal plane^60^, it also necessitates some leg joint flexion in the sagittal plane to raise the leading and trailing legs and to provide a foot-strike and push-off impulse to achieve a step-to-step transition. Yet, compared to children with CP, these angular joint movements in the sagittal plane are comparatively small in TD children (Fig. 4).

While almost all TD children (except for the youngest child, Fig. 5A left panel) performed the task in an adult-like way (i.e., the leading leg was abducting and the trailing leg adducting such that the trailing limb did not cross ahead of the leading limb), children with CP often demonstrated attempts to step forward while walking sideways. In particular, they tended to rotate their trunk (Fig. 5B), cross one leg over the other (Fig. 5A), generate a hip flexion torque (Fig. 4B) and flex the knee during the swing phase (Fig. 4A). Such directional movements and attempts to move forward were observed in most children with CP independent of age and despite the fact that, prior to the recordings, the experimenter herself/himself showed movements that the child had to perform. Before to the trial, the children did not practice SW, and we recorded a limited number of strides from each child (Table 2). Nevertheless, even if we did not study potential effects of practice in SW in CP, attempts to rotate the trunk were observed throughout the whole experiment, across repeated strides and trials. Probably, a normal mature-like performance of SW (without elements of forward stepping) requires learning to step sideways and/or performing earlier treatment of adaptive gait in children with CP considering critical developmental windows in maturation of supraspinal pathways and gait control^3,4,6^.

### Adjustments in the spinal locomotor output and interlimb coordination during SW

Why did children with cerebral palsy tend to step forward while they moved sideways? Possibly, femoral deformities, somewhat flexed posture, or pelvis stability in children with CP may contribute to the difficulties in performing SW. Also, one cannot rule out the impact of distal muscle spasticity, since ankle plantarflexors assist in generating a foot-strike and push-off impulse, and their spasticity could possibly make participants more likely to step forward during SW. Children with CP, for instance, who failed to perform the SW task displayed some degree of spasticity (Table 2). Nevertheless, children who were successful at this task also demonstrated spasticity (Table 2) and we did not find a strong relationship between the MAS scores and the percentage of steps with 5MTtr_x > 0 (crossing one foot over the other) or trunk yaw rotations (Fig. 5). In addition, the attempts to step forward while moving sideways in children with CP were associated with augmented torques in the proximal (hip) leg joint rather with the moments of force in distal extensors (Fig. 4). Therefore, it is unlikely that plantarflexor spasticity is an essential or the only contributing factor. Our examination of the spinal locomotor output (Figs. 6, 7), however, raises the possibility that the neural mechanisms of the interlimb coordination may have a significant impact on the failure of adaptive gait regulation.

There is a growing interest in the neural mechanisms of adaptive locomotion, their development, and the role of supraspinal structures in controlling spinal pattern generators. The multidirectional locomotor tasks (backward, sideways), that most mammals are able to perform in the context of avoidance behavior, accommodation to different environmental contexts, and postural corrections^61^, represent biomechanically distinct cyclic movements that share some (not all) common neural control pathways^48,62^. In particular, the adaptive control is consistent with the idea of unit rhythmic pattern generators for each limb, joint, or groups of muscles that can be combined in a flexible way to provide various gait patterns^63^. Investigating functional organization of neural circuits reveals the common rhythm-generating part of locomotor networks, while networks determining direction of progression may be specific for each direction^61^. However, both backward and sideways locomotion may well depend more on supraspinal, especially cortical, inputs than more standard forward locomotion.

Stepping sideways is distinctive in that it necessitates asymmetrical coordination and interaction between locomotor pattern generators of the left and right limbs, in contrast to other changes in direction of progression (such as walking backward, uphill, downhill). The role of each limb during SW can be assessed by examining the spatiotemporal organization and coordination of activity of bilateral muscles, given that SW requires a differential control of the left and right limb flexor and extensor burst generators to perform this biomechanically asymmetrical task. In TD children during SW, the muscle activity of the leading and trailing limbs was significantly different; in particular, the leading limb's BF, ST, MG, LG, SOL, and TA activity displayed systematic variations in relation to FW. For instance, the leading limb's TA activity shifted toward mid-stance, whereas the calf muscles' activity was clearly shifted toward the end of the swing and the beginning of stance. In children with CP, the activity of these muscles paralleled that of FW, so that these interlimb variations were absent or less noticeable (Fig. 6). In line with individual muscle activity adjustments, the basic patterns of the 1st and 3rd activation modules (Fig. 7A), which are linked to muscle synergies involving distal muscles, were quite similar between FW and SW in TD children, whereas the basic patterns of the 2nd and 4th modules, associated with proximal muscles, were "adaptable" to SW. Conversely, due to the closeness of the c2 and c4 activation patterns during SW with the equivalent patterns during FW, the same basic activation patterns did not display the same modifications from FW to SW in children with CP. These findings show that children with CP, who remarkably displayed similar motor modules for FW and SW, lack adaptations (Figs. 6, 7).

Whatever the exact mechanisms behind the interlimb coordination changes, the challenges faced by children with CP when walking SW reflect the general developmental deficit in adaptive locomotor behaviour^9,10^. For the primary form of locomotion (FW), where some features of independent control of each leg have been documented^10,64^, the multidirectional tasks (such as SW) probably present a higher barrier for children with CP. Most children adapted to this task and managed to step sideways (Table 2), however the analysis of the spatiotemporal muscle activity patterns clearly revealed the lack of flexibility in the task-relevant muscle activity control. The major difference we found in the performance of SW between CP and TD children is that there was a clear differentiation of the muscle activation modules between the leading and trailing limbs in TD children, whereas there was a lack of adjustments in children with CP (Figs. 6, 7).

## Concluding remarks

Whereas the impairments of forward gait and its unsteadiness have been extensively investigated in children with CP, the neural mechanisms of the adaptive locomotor behavior have been studied to a lesser extent. The remarkable feature of CP gait is the reduced flexibility in the differential control of the left and right leg unit burst generators (Figs. 6, 7). These results corroborate previous findings on the impaired performance of adaptive locomotion and a lack of flexibility in adjustment of basic locomotor modules to the specific task^9,10^. For instance, limited adjustments of task-relevant activity of hamstring muscles timed to the voluntary task of foot lift were also observed during stepping over an obstacle in children with CP^10^. Lack of flexibility and impaired task performance in children with CP may reflect basic developmental deficits in the adaptable control of gait, suggesting that gait rehabilitation strategies should involve challenging directional tasks to enhance the functional capacity and flexibility of gait controllers.

## Data Availability

The datasets used in the current study are available from the corresponding author on reasonable request.

## References

[1] Adolph, K. E. & Robinson, S. R. Motor development. In *Handbook of Child Psychology and Developmental Science: Cognitive Processes*, vol. 2, 7th ed 113–157 (Wiley, 2015). 10.1002/9781118963418.childpsy204.

[2] Hadders-Algra M (2018). Early human motor development: From variation to the ability to vary and adapt. Neurosci. Biobehav. Rev..

[3] Cappellini G (2020). Maturation of the locomotor circuitry in children with cerebral palsy. Front. Bioeng. Biotechnol..

[4] Friel KM, Williams PTJA, Serradj N, Chakrabarty S, Martin JH (2014). Activity-based therapies for repair of the corticospinal system injured during development. Front. Neurol..

[5] Hadders-Algra M (2004). General movements: A window for early identification of children at high risk for developmental disorders. J. Pediatr..

[6] Hurd C (2017). Early intensive leg training to enhance walking in children with perinatal stroke: Protocol for a randomized controlled trial. Phys. Ther..

[7] Ritterband-Rosenbaum A (2017). A critical period of corticomuscular and EMG–EMG coherence detection in healthy infants aged 9–25 weeks. J. Physiol..

[8] Morgan C (2021). Early intervention for children aged 0 to 2 years with or at high risk of cerebral palsy: International clinical practice guideline based on systematic reviews. JAMA Pediatr..

[9] Cappellini G (2018). Backward walking highlights gait asymmetries in children with cerebral palsy. J. Neurophysiol..

[10] Cappellini G (2020). Locomotor patterns during obstacle avoidance in children with Cerebral Palsy. J. Neurophysiol..

[11] Graham HK (2016). Cerebral palsy. Nat. Rev. Dis. Primers.

[12] Meyns P (2016). Macrostructural and microstructural brain lesions relate to gait pathology in children with cerebral palsy. Neurorehabil. Neural Repair.

[13] Abdel-Aziem AA, El-Basatiny HM (2017). Effectiveness of backward walking training on walking ability in children with hemiparetic cerebral palsy: A randomized controlled trial. Clin. Rehabil..

[14] Kim W-H, Kim W-B, Yun C-K (2016). The effects of forward and backward walking according to treadmill inclination in children with cerebral palsy. J. Phys. Ther. Sci..

[15] Malone A, Kiernan D, French H, Saunders V, O’Brien T (2016). Obstacle crossing during gait in children with cerebral palsy: Cross-sectional study with kinematic analysis of dynamic balance and trunk control. Phys. Ther..

[16] Malone A, Kiernan D, French H, Saunders V, O’Brien T (2015). Do children with cerebral palsy change their gait when walking over uneven ground?. Gait Posture.

[17] Meyns P (2016). Children with spastic cerebral palsy experience difficulties adjusting their gait pattern to weight added to the waist, while typically developing children do not. Front. Hum. Neurosci..

[18] Lacquaniti F, Ivanenko YP, Zago M (2012). Development of human locomotion. Curr. Opin. Neurobiol..

[19] Yang JF, Mitton M, Musselman KE, Patrick SK, Tajino J (2015). Characteristics of the developing human locomotor system: Similarities to other mammals. Dev. Psychobiol..

[20] Dewolf AH, Sylos Labini F, Ivanenko Y, Lacquaniti F (2020). Development of locomotor-related movements in early infancy. Front. Cell Neurosci..

[21] Patrick SK, Noah JA, Yang JF (2012). Developmental constraints of quadrupedal coordination across crawling styles in human infants. J. Neurophysiol..

[22] Sylos-Labini F (2020). Distinct locomotor precursors in newborn babies. Proc. Natl. Acad. Sci. U.S.A..

[23] Adolph KE, Berger SE, Leo AJ (2011). Developmental continuity? Crawling, cruising, and walking. Dev. Sci.

[24] Dewolf, A. H., Sylos-Labini, F., Cappellini, G., Lacquaniti, F. & Ivanenko, Y. Emergence of different gaits in infancy: Relationship Between developing neural circuitries and changing biomechanics. *Front. Bioeng. Biotechnol.* **8**, (2020). 10.3389/fbioe.2020.00473 PMC7248179 32509753

[25] Zelik KE, La Scaleia V, Ivanenko YP, Lacquaniti F (2014). Can modular strategies simplify neural control of multidirectional human locomotion?. J. Neurophysiol..

[26] Rethlefsen SA, Blumstein G, Kay RM, Dorey F, Wren TAL (2017). Prevalence of specific gait abnormalities in children with cerebral palsy revisited: influence of age, prior surgery, and Gross Motor Function Classification System level. Dev. Med. Child Neurol..

[27] Kuntze G, Sellers WI, Mansfield N (2009). Bilateral ground reaction forces and joint moments for lateral sidestepping and crossover stepping tasks. J. Sports Sci. Med..

[28] Dewolf AH (2022). Left-right locomotor coordination in human neonates. J. Neurosci..

[29] Yang JF, Lamont EV, Pang MYC (2005). Split-belt treadmill stepping in infants suggests autonomous pattern generators for the left and right leg in humans. J. Neurosci..

[30] Himmelmann K, Hagberg G, Beckung E, Hagberg B, Uvebrant P (2005). The changing panorama of cerebral palsy in Sweden. IX. Prevalence and origin in the birth-year period 1995–1998. Acta Paediatr..

[31] Palisano R (1997). Development and reliability of a system to classify gross motor function in children with cerebral palsy. Dev. Med. Child Neurol..

[32] Palisano RJ, Cameron D, Rosenbaum PL, Walter SD, Russell D (2006). Stability of the gross motor function classification system. Dev. Med. Child Neurol..

[33] Hidecker MJC (2011). Developing and validating the Communication Function Classification System for individuals with cerebral palsy. Dev. Med. Child Neurol..

[34] Russell DJ (1989). The gross motor function measure: A means to evaluate the effects of physical therapy. Dev. Med. Child Neurol..

[35] Russell DJ (2000). Improved scaling of the gross motor function measure for children with cerebral palsy: Evidence of reliability and validity. Phys. Ther..

[36] Bohannon RW, Smith MB (1987). Interrater reliability of a modified Ashworth scale of muscle spasticity. Phys. Ther..

[37] Dominici N, Ivanenko YP, Lacquaniti F (2007). Control of foot trajectory in walking toddlers: Adaptation to load changes. J. Neurophysiol..

[38] Cappellini G (2016). Immature spinal locomotor output in children with cerebral palsy. Front. Physiol..

[39] Winter, D. A. *Biomechanics and Motor Control of Human Movement*. (Wiley, 2009).

[40] Dempster, W. T. *Space Requirements of the Seated Operator, Geometrical, Kinematic, and Mechanical Aspects of the Body with Special Reference to the Limbs*. (1955).

[41] Martino G (2015). Neuromuscular adjustments of gait associated with unstable conditions. J. Neurophysiol..

[42] Torres-Oviedo G, Macpherson JM, Ting LH (2006). Muscle synergy organization is robust across a variety of postural perturbations. J. Neurophysiol..

[43] Shuman BR, Goudriaan M, Desloovere K, Schwartz MH, Steele KM (2019). Muscle synergies demonstrate only minimal changes after treatment in cerebral palsy. J. Neuroeng. Rehabil..

[44] Steele KM, Munger ME, Peters KM, Shuman BR, Schwartz MH (2019). Repeatability of electromyography recordings and muscle synergies during gait among children with cerebral palsy. Gait Posture.

[45] Yu Y (2019). Gait synergetic neuromuscular control in children with cerebral palsy at different gross motor function classification system levels. J. Neurophysiol..

[46] Saltiel P, Wyler-Duda K, D’Avella A, Tresch MC, Bizzi E (2001). Muscle synergies encoded within the spinal cord: Evidence from focal intraspinal NMDA iontophoresis in the frog. J. Neurophysiol..

[47] Batschelet, E. *Circular Statistics in Biology*. (Academic Press, 1981).

[48] Lamb T, Yang JF (2000). Could different directions of infant stepping be controlled by the same locomotor central pattern generator?. J. Neurophysiol..

[49] Damiano DL, Martellotta TL, Sullivan DJ, Granata KP, Abel MF (2000). Muscle force production and functional performance in spastic cerebral palsy: Relationship of cocontraction. Arch. Phys. Med. Rehabil..

[50] Prosser LA, Lee SCK, VanSant AF, Barbe MF, Lauer RT (2010). Trunk and hip muscle activation patterns are different during walking in young children with and without cerebral palsy. Phys. Ther..

[51] Steele KM, Rozumalski A, Schwartz MH (2015). Muscle synergies and complexity of neuromuscular control during gait in cerebral palsy. Dev. Med. Child Neurol..

[52] Shuman B (2016). Repeatability of muscle synergies within and between days for typically developing children and children with cerebral palsy. Gait Posture.

[53] Goudriaan M (2022). Muscle synergy structure and gait patterns in children with spastic cerebral palsy. Dev. Med. Child. Neurol..

[54] d’Avella A, Ivanenko Y, Lacquaniti F (2022). Muscle synergies in cerebral palsy and variability: Challenges and opportunities. Dev. Med. Child Neurol..

[55] Drew T, Andujar J-E, Lajoie K, Yakovenko S (2008). Cortical mechanisms involved in visuomotor coordination during precision walking. Brain Res. Rev..

[56] Drew T, Marigold DS (2015). Taking the next step: Cortical contributions to the control of locomotion. Curr. Opin. Neurobiol..

[57] Fazzi E (2009). Cognitive visual dysfunctions in preterm children with periventricular leukomalacia. Dev. Med. Child Neurol..

[58] Lajoie K, Andujar J-E, Pearson K, Drew T (2010). Neurons in area 5 of the posterior parietal cortex in the cat contribute to interlimb coordination during visually guided locomotion: A role in working memory. J. Neurophysiol..

[59] Sansare A, Arcodia M, Lee SCK, Jeka J, Reimann H (2022). Individuals with cerebral palsy show altered responses to visual perturbations during walking. Front. Hum. Neurosci..

[60] Handford ML, Srinivasan M (2014). Sideways walking: Preferred is slow, slow is optimal, and optimal is expensive. Biol. Lett..

[61] Deliagina TG, Musienko PE, Zelenin PV (2019). Nervous mechanisms of locomotion in different directions. Curr. Opin. Physiol..

[62] Hoogkamer W, Meyns P, Duysens J (2014). Steps forward in understanding backward gait: From basic circuits to rehabilitation. Exerc. Sport Sci. Rev..

[63] Grillner S, El Manira A (2020). Current principles of motor control, with special reference to vertebrate locomotion. Physiol. Rev..

[64] Bulea TC, Stanley CJ, Damiano DL (2017). Part 2: Adaptation of gait kinematics in unilateral cerebral palsy demonstrates preserved independent neural control of each limb. Front. Hum. Neurosci..

